# The combined effects of reactant kinetics and enzyme stability explain the temperature dependence of metabolic rates

**DOI:** 10.1002/ece3.2955

**Published:** 2017-04-23

**Authors:** J. P. DeLong, J. P. Gibert, T. M. Luhring, G. Bachman, B. Reed, A. Neyer, K. L. Montooth

**Affiliations:** ^1^ School of Biological SciencesUniversity of Nebraska – LincolnLincolnNEUSA; ^2^ Present address: The University of California, MercedMercedCAUSA

**Keywords:** acclimation, metabolic rate, thermal adaptation, thermal performance curve

## Abstract

A mechanistic understanding of the response of metabolic rate to temperature is essential for understanding thermal ecology and metabolic adaptation. Although the Arrhenius equation has been used to describe the effects of temperature on reaction rates and metabolic traits, it does not adequately describe two aspects of the thermal performance curve (TPC) for metabolic rate—that metabolic rate is a unimodal function of temperature often with maximal values in the biologically relevant temperature range and that activation energies are temperature dependent. We show that the temperature dependence of metabolic rate in ectotherms is well described by an enzyme‐assisted Arrhenius (EAAR) model that accounts for the temperature‐dependent contribution of enzymes to decreasing the activation energy required for reactions to occur. The model is mechanistically derived using the thermodynamic rules that govern protein stability. We contrast our model with other unimodal functions that also can be used to describe the temperature dependence of metabolic rate to show how the EAAR model provides an important advance over previous work. We fit the EAAR model to metabolic rate data for a variety of taxa to demonstrate the model's utility in describing metabolic rate TPCs while revealing significant differences in thermodynamic properties across species and acclimation temperatures. Our model advances our ability to understand the metabolic and ecological consequences of increases in the mean and variance of temperature associated with global climate change. In addition, the model suggests avenues by which organisms can acclimate and adapt to changing thermal environments. Furthermore, the parameters in the EAAR model generate links between organismal level performance and underlying molecular processes that can be tested for in future work.

## 1 Introduction

Temperature plays a major role in setting biological rates across all levels of organization, from biochemical reactions within cells to nutrient turnover in ecosystems (Brown, Gillooly, Allen, Savage, & West, 2004; Hochachka & Somero, 2002; Kleiber, 1961; Schulte, 2015; Yvon‐Durocher et al., 2012). The temperature dependence of metabolic rate is among the most fundamental of thermal relationships, playing a significant role in setting the temperature dependence of many other biological processes (Brown et al., 2004; Dell, Pawar, & Savage, 2011; Gillooly, Brown, West, Savage, & Charnov, 2001). As such, an understanding of the response of metabolic rate to temperature is essential for understanding thermal ecology.

**Figure 1:**
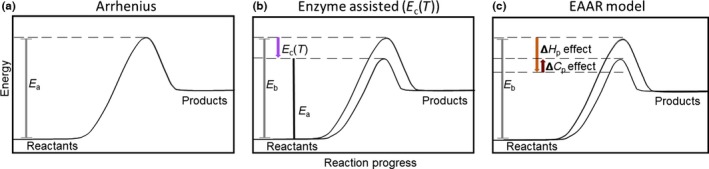
(a) Reactions proceed as the reactants gain enough energy to clear the hurdle of the activation energy (*E*_*a*_) to form products. (b) Organisms contribute some energy to reactions occurring within their bodies with enzymes. The contributed energy lowers the kinetic hurdle that reactants must clear, such that the net activation energy is *E*_*b*_ − *E*_*c*_(*T*), the latter of which is temperature dependent via effects of temperature on protein stability. (c) Metabolic reactions have to clear their particular activation energy (*E*_*b*_, gray bar), but enzymes provide a temperature‐dependent contribution to the starting energetic state of the reactants through a temperature‐dependent increase in stability (ΔH, orange arrow). Below the melting temperature, however, temperature lowers the energetic state through its effect on heat capacity (ΔCp, red arrow)

The metabolic rate of ectotherms typically increases rapidly as temperature increases from lower temperatures, and this increase is often described using an Arrhenius function (Dell et al., 2011; Gillooly et al., 2001; Robinson, Peters, & Zimmermann, 1983). The Arrhenius equation models the effect of temperature on the rate (*V*) of a reaction by scaling the potential reaction rate *A*_0_ (set by the availability of appropriately conformed reactants) by the Arrhenius factor, $e^{-E_{a}/kT}$, where *k* is Boltzmann's constant (8.61 × 10^−5^ eV K^−1^), *T* is absolute temperature (K), and *E*_*a*_ is the activation energy—the minimum energy that must be available for the chemical reaction to occur (eV) (Figure 1a; Laidler, 1984):

(1) $$V=A_{0}e^{-E_{a}/kT},$$

The Arrhenius factor varies between 0 and 1 (Figure S1), giving the proportion of the potential reaction rate *A*_0_ that can occur given the kinetic state of the reactants. The product *kT* is the average kinetic energy of the reactants, such that as temperature increases, the energy of the reactants increases, reducing the value of the exponent and raising $e^{-E_{a}/kT}$ toward 1.

The Arrhenius equation was originally applied to describe the temperature dependence of chemical reaction rates in controlled settings, but it has also been applied to describe the thermal dependence of biological rates, including enzyme‐catalyzed reactions and organismal metabolic rate, an application widely promoted in the metabolic theory of ecology (MTE; Brown et al., 2004). Despite the good fit of the Arrhenius equation to many data sets and its widespread use by ecologists (Allen, Brown, & Gillooly, 2002; Anderson‐Teixeira, DeLong, Fox, Brese, & Litvak, 2010; Dell et al., 2011; Ernest et al., 2003; López‐Urrutia, San Martin, Harris, & Irigoien, 2006; O'Connor, Piehler, Leech, Anton, & Bruno, 2009; Yvon‐Durocher et al., 2012), there are two unresolved problems with using the Arrhenius equation to describe the temperature dependence of metabolic rate. First, the Arrhenius factor is a monotonically increasing function of temperature, whereas metabolic rate and many other biological rates are unimodal functions of temperature, generally known as thermal performance curves (TPC; Huey & Stevenson, 1979; Huey & Kingsolver, 1989; Angilletta, Niewiarowski, & Navas, 2002). It is often argued that the Arrhenius equation is sufficient because it applies to the range of temperatures sometimes referred to as the “biologically relevant temperature range” (usually 0–40°C; Figure S1) or, alternatively, the temperature range between the minimum temperature and the temperature at which maximal metabolic rates are observed (*T*_opt_) (Gillooly et al., 2001). The justification for focusing on restricted temperature ranges is that these are the temperatures at which organisms spend most of their time. Although this may be true in some cases, many organisms experience temperatures above their *T*_opt_, where metabolic rates decrease and thus are not expected to be well described by the Arrhenius equation, as the Arrhenius factor increases monotonically with temperature. This problem is currently becoming more important, as future climate scenarios predict warmer and more variable temperatures (Schulte, 2015), causing organisms to spend more time at the upper extremes of their viable temperature ranges where the Arrhenius equation does not apply, or, if used, would overestimate metabolic rates.

Second, the activation energy is a constant in the standard Arrhenius model, yet activation energy can vary across biologically relevant temperatures (Gibert, Chelini, Rosenthal, & DeLong, 2016; Knies & Kingsolver, 2010; Pawar, Dell, Savage, & Knies, 2016; Schulte, 2015). Such empirical observations suggest that even over temperature ranges where the Arrhenius equation is thought to apply, it may not be sufficiently nuanced to enable prediction of metabolic responses to temperature under warmer and more variable climates (Gibert et al., 2016; Pawar et al., 2016).

There have been several efforts to modify the Arrhenius equation and generate models that describe the unimodal response of reaction rates to temperature (Box 1). Beginning with Johnson and Lewin (1946), at least five models have modified an Arrhenius (or the similar Eyring) function by discounting the rates at low and/or high temperatures (Daniel & Danson, 2010; Ratkowsky, Olley, & Ross, 2005; Schoolfield, Sharpe, & Magnuson, 1981; Sharpe & DeMichele, 1977). These models use different functions to reduce the probability of enzymes being in an active state at low and/or high temperatures, but are consistent in the assumption that the activation energy in the Arrhenius type function corresponds to the state of maximal enzyme activity (Box 1, Assumption 10, Figure B 1). This assumption is problematic because it requires that in the absence of enzymes, the reaction would still occur with a low activation energy corresponding to fully active enzymes. Furthermore, this assumption contradicts what enzymes are actually thought to do, which is to lower the activation energy below a baseline level (Box 1, Assumption 12). The more recent macromolecular rates model (Hobbs et al., 2013) uses a different approach, altering the activation of a reaction directly through a protein stability curve. Although this approach shows promise, there are three problematic assumptions with this model. First, the activation energy of the reaction is made equal to the free energy of the enzymes themselves (Box 1, Assumption 8). These energies are not the same, as recognized by the previous models that separate out an activation energy for a reaction from the free energy of the catalysts (Box 1). Second, the model requires the heat capacity of the catalysts to be negative (Box 1, Assumption 9), when this value must be positive, as indicated in previous work (Becktel & Schellman, 1987; Feller, 2010; Ratkowsky et al., 2005). Third, the model reduces to a linear function of temperature in the absence of enzymes (Box 1, Assumption 11) with a universal slope of the Boltzmann constant divided by Plank's constant, rather than an Arrhenius type function. For these and other reasons (see key assumptions in Box 1), the current unimodal modifications of the Arrhenius function are not sufficient.

Box 1 **A history of models describing the unimodal temperature dependence of enzyme‐catalyzed reaction rates**

**Figure B1:**
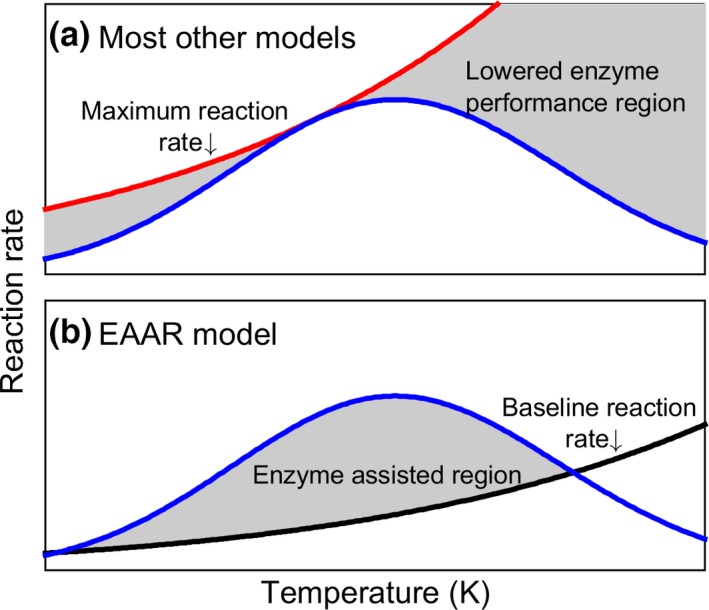
How the EAAR model and other models bend Arrhenius type functions to create a unimodal function. (a) Most models assume a maximal reaction rate (red line) that is discounted by lowered probability of enzyme performance, reducing the reaction rate to the blue line. Note that model 1 has a lowered performance region only at high temperature. (b) The EAAR model begins with the assumption that there is a physical baseline reaction rate (black line) that may occur outside of an organism and that has a relatively high activation energy. Inside an organism, enzymes assist the process by lowering the activation energy, boosting the reaction rate above what would otherwise be expected, to the blue line

1 A range of models have been developed to describe the dependence of a reaction rate (*V*, for reaction velocity) on temperature (*T*). Most of these models were originally developed to describe enzyme‐catalyzed reactions, rather than metabolic rate *per se*, but model 4 was developed to describe population growth rate and model 3 originally dealt with development rate. Nonetheless, they all have potential as descriptions of the temperature dependence of metabolic rate. All of the models begin with a monotonically increasing function of temperature, either the Eyring or the Arrhenius equation, the difference being that the Eyring model explicitly includes temperature in the constant. All of the models invoke a reduction in enzyme performance at low and/or high temperature due to the decreased probability of enzymes being in an active state. A key difference between the EAAR model and models 1‐5 is that in the EAAR model, enzymes increase reaction rates over some baseline rate, while in models 1‐5, reduced enzyme performance lowers the reaction rate from a maximal rate (Figure B1).

The models invoke different assumptions, some of which are shared across models and others which are unique to specific models. The key assumptions invoked by each model are indicated with a check‐mark in the Table and listed here: (1) enzymes are inactive at high temperature, (2) enzymes are inactive at low temperature, (3) active state is given by a three‐state transition process, (4) active state is given by a protein stability process, (5) substrate supply is unlimited, (6) the proportion of enzymes that are in an active state is at equilibrium, (7) enzymes denature through time, (8) the activation energy of the catalyzed reaction is equal to the free energy of the catalyzing enzymes, (9) heat capacity is negative, (10) activation energy corresponds to maximal enzyme activity level, (11) there is no activation energy in the absence of enzymes, (12) the activation energy of the reaction is lowered as a function of the free energy of the catalyzing enzymes.

The common parameters in these models are Δ*H*, enthalpy change of folding the enzymes, relative to a reference or melting temperature, subscripted *A* for active state and *L* for lower temperature inactive state; Δ*S*, entropy change of an enzyme with temperature, relative to the melting temperature, subscripted *A* for active state and *L* for lower temperature inactive state; Δ*C*_*p*_, the difference in heat capacity between the folded and unfolded state of the enzymes, relative to the melting temperature; *T*_*m*_, melting temperature. The Schoolfield model includes three summary parameters: ρ_(25°C)_, rate at 25°C assuming no inactivation; T_1/2_, the temperature at which the enzyme is half active and half inactive, subscripted *L* for low‐temperature inactive and *H* for high‐temperature inactive. The equilibrium model includes four parameters: *T*_eq_, the temperature at which the concentration of active and inactive enzymes is equal; Δ*H*_eq_, the change in enthalpy associated with the equilibrium; Δ*G*_cat_, activation energy for the reaction; and Δ*G*_inact_, activation energy for enzyme inactivation. The EAAR model introduces *E*_*b*_, the baseline activation energy, the change in activation energy associated with the change in enthalpy of the catalysts (*E*_Δ*Cp*_), and the change in activation energy associated with the change in heat capacity of the catalysts (*E*_Δ*H*_). The physical constants are *k*, Boltzmann's constant, *R*, the gas constant, and *h*, Planck's constant.

**Table:** 

Model #	Model/source	Model	Assumptions											
			1	2	3	4	5	6	7	8	9	10	11	12
1	Johnson‐Lewin (Johnson & Lewin, 1946)	$V=\frac{cTe^{\Delta H_{A}/RT}}{1+e^{\Delta S/R}e^{\Delta H_{A}/RT}}$	✔				✔					✔		
2	Sharpe‐DeMichele (Sharpe & DeMichele, 1977)	$V=\frac{\frac{kT}{h}e^{(\Delta S_{A}-\Delta H_{A}/T)/R}}{1+e^{(\Delta S_{L}-\Delta H_{L}/T)/R}+e^{(\Delta S_{H}-\Delta H_{L}/T)/R}}$	✔	✔	✔		✔					✔		
3	Schoolfield (Schoolfield et al., 1981)	$V=\frac{\rho_{\left(25{\;}^{\circ }C\right)}\frac{T}{298}e^{\left[\frac{\Delta H_{A}}{R}\left(\frac{1}{298}-\frac{1}{T}\right)\right]}}{1+e^{\left[\frac{\Delta H_{L}}{R}\left(\frac{1}{T_{1/2L}}-\frac{1}{T}\right)\right]}+e^{\left[\frac{\Delta H_{H}}{R}\left(\frac{1}{T_{1/2H}}-\frac{1}{T}\right)\right]}}$	✔	✔	✔		✔					✔		
4	Ratkowsky (Ratkowsky et al., 2005)	$V=\frac{cTe^{\left(\Delta H_{A}/RT\right)}}{1+e^{\left(-n[\Delta H-T\Delta S+\Delta C_{p}[\left(T-T_{m}\right)-T\text{ln}\left(T/T_{m}\right)]\right)]/RT)}}$	✔	✔		✔	✔					✔		
5	Equilibrium model (Daniel & Danson, 2010)	$V=\frac{kT}{h}\frac{e^{-(\Delta G_{cat}/RT)}E_{0}e^{\left[\frac{kTe^{-(\Delta G_{inact}/RT)}e^{\left(\Delta H_{eq}\left(\frac{1}{T_{eq}}-\frac{1}{T}\right)/R\right)}t}{1+e^{\left(\Delta H_{eq}\left(\frac{1}{T_{eq}}-\frac{1}{T}\right)/R\right)}}\right]}}{1+e^{\left(\Delta H_{eq}\left(\frac{1}{T_{eq}}-\frac{1}{T}\right)/R\right)}}$	✔				✔	✔	✔			✔		
6	Macromolecular rates (Hobbs et al., 2013)	$V=\frac{kT}{h}e^{\frac{-\left(\Delta H-T\Delta S+\Delta C_{p}\left(T-T_{m}-T\text{ln}\frac{T}{T_{m}}\right)\right)}{RT}}$	✔	✔		✔				✔	✔		✔	
7	Enzyme‐assisted Arrhenius (This study)	$V=A_{0}e^{\frac{-\left(E_{b}-\left(E_{\Delta H}\left(1-\frac{T}{T_{m}}\right)+E_{\Delta Cp}\left(T-T_{m}-T\text{ln}\frac{T}{T_{m}}\right)\right)\right)}{kT}}$	✔	✔		✔								✔

A model that describes and predicts metabolic rate TPCs based on more realistic biological mechanisms is therefore urgently needed (Schulte, 2015). Here, we derive a general model for the temperature dependence of metabolic rate with biologically meaningful parameters that captures the unimodal shape of a metabolic rate TPC. We do this by incorporating a mechanistically derived temperature‐dependent protein stability curve, which specifies the extent to which enzymes can catalyze metabolic reactions, into the Arrhenius equation to capture the temperature dependence in the ability of enzymes to lower activation energies, yielding the enzyme‐assisted Arrhenius (EAAR) model.

## 2 What is the Arrhenius equation missing?

Within an organism, biological reactions are assisted by enzymes that connect reactants in a spatially appropriate way and lower the kinetic energy needed for the reaction to proceed. It is generally appreciated that the *E*_*a*_ in the Arrhenius equation, as it is used to describe biological rates, will be set by enzymes. However, it is more precise to say that the realized *E*_*a*_ represents the difference between the kinetic requirements of a reaction as it would occur outside of an organism (i.e., without catalysts, the baseline energy, *E*_*b*_) and the enzymatic contribution (*E*_*c*_) of the organism to the reaction (Figure 1b). We can therefore rewrite the Arrhenius equation to explicitly include both the baseline energy and the energetic contribution of enzymes to the process:

(2) $$V=A_{0}e^{\frac{-\left(E_{b}-E_{c}\right)}{kT}},$$

The observed activation energy (*E*_*a*_), then, is the kinetic hurdle that remains after enzymes have done their job (*E*_*b*_ − *E*_*c*_).

What is missing from the Arrhenius model is a recognition that the activity level of enzymes follows a hump‐shaped relation with temperature (Feller, 2010; Peterson, Daniel, Danson, & Eisenthal, 2007), as recognized in previous unimodal models (Box 1). At low and/or high temperatures, enzymes may occur in inactive states, either through reversible unfolding or denaturation. As a consequence, enzymes are less effective at reducing the *E*_*b*_ of metabolic rate at low and high temperatures. Thus, increasing enzymatic contributions with increasing temperature helps to increase the metabolic rate up to the *T*_opt_, while decreasing enzymatic contributions as temperature continues to increase generates the decreasing slope observed for metabolic rate TPCs.

Here, we incorporate a model for protein stability/free energy into the Arrhenius equation to provide a mechanistic basis for the temperature dependence of metabolic rate that accounts for the contributions of both reactant kinetics and the temperature dependence of enzyme activity. This is an important conceptual advancement over our current description of the rising portion of metabolic rate TPCs as a function of the energetic state of reactants. This change in viewpoint clarifies how and why activation energies should change during the rising portion of the TPC, and why metabolic rate should decline again above an optimal temperature.

## 3 The enzyme‐assisted Arrhenius model (*EAAR*)

Biochemical reactions within organisms require sufficient kinetic activation and the catalytic contribution of enzymes (Segel, 1975). Protein stability curves depict the ΔG (change in Gibbs free energy, kcal/mol or equivalently in eV) between the folded and unfolded states as a function of temperature, or the amount of work that must be done to induce a transition in a protein from the folded to the unfolded state at each temperature (Haynie, 2008). Mechanistic derivations of protein stability curves indicate that the temperature dependence of ΔG follows a hump‐shaped function of temperature (Becktel & Schellman, 1987; Feller, 2010):

(3) $$\Delta G=\Delta H\left(1-\frac{T}{T_{m}}\right)+\Delta C_{p}\left(T-T_{m}-T\text{ln}\frac{T}{T_{m}}\right),$$

where Δ*H* is the enthalpy of folding the enzymes used in the metabolic reaction, relative to the melting temperature, *T*_*m*_, and Δ*C*_*p*_ is the difference in heat capacity between the folded and unfolded state of the enzymes, again relative to the melting temperature. ΔG reflects the stability of the enzyme, and critically, the probability of an enzyme being in an active state and thus its ability to lower the activation energy of the reaction (Feller, 2010; Hobbs et al., 2013; Ratkowsky et al., 2005). As indicated above, the free energy of the catalyst is not equal to the reduction in the activation energy. Rather, the probability that enzymes are in an active state approaches 1 at the maximum ΔG. We therefore divide Equation (3) by ΔG_max_ to transform it into a probability. Given that it is in an active state, the catalyst lowers the activation by an amount *E*_*L*_, such that $E_{c}=E_{L}\frac{\Delta G}{\Delta G_{\max}}$. Thus, we replace each parameter in Equation (3) to account for this transformation (i.e., $E_{\Delta H}=E_{L}\frac{\Delta H}{\Delta G_{\max}}$, $E_{\Delta Cp}=E_{L}\frac{\Delta C_{p}}{\Delta G_{\max}}$) and rewrite Equation (3) as follows:

(4) $$E_{c}=E_{\Delta H}\left(1-\frac{T}{T_{m}}\right)+E_{\Delta Cp}\left(T-T_{m}-T\text{ln}\frac{T}{T_{m}}\right)$$

Substituting Equation (4) into Equation (2), we get

(5) $$V=A_{0}e^{\frac{-\left(E_{b}-\left(E_{\Delta H}\left(1-\frac{T}{T_{m}}\right)+E_{\Delta Cp}\left(T-T_{m}-T\text{ln}\frac{T}{T_{m}}\right)\right)\right)}{kT}},$$

which now provides a mechanistic description of the temperature dependence of metabolic rate that is generated by both reactant kinetics and temperature‐dependent enzyme stability.

The thermodynamic parameters in Equation (5) provide a nonphenomenological depiction of how enzyme stability alters reaction rates. Δ*H* is by definition zero at the melting point and increases below the melting point, meaning that the colder it gets, the more stable the enzyme is and the more effectively it can contribute to a reaction (Figure 1c). Δ*C*_*p*_ scales the loss of free energy as temperature goes below the melting point, meaning the colder it gets below the melting temperature, the more energy the enzyme can retain without changing temperature, in effect scrubbing free energy from the system. The expression $\left(T-T_{m}-T\text{ln}\frac{T}{T_{m}}\right)$ thus represents the loss of function associated with being unfolded and is zero at the melting temperature. The parameters in our model reflect the change in activation energy associated with the change in enthalpy of the catalysts (*E*_Δ*Cp*_) and the change in activation energy associated with the change in heat capacity of the catalysts (*E*_Δ*H*_).

Taking the derivative of Equation (5) with respect to temperature and then rearranging terms provides an explicit expression for the optimal temperature (*T*_opt_), where the top of the unimodal TPC is reached:

(6) $$T_{opt}=\frac{E_{b}-E_{\Delta H}+E_{\Delta Cp}T_{m}}{E_{\Delta Cp}}.$$

This expression shows that modifying *T*_opt_ through acclimation or adaptation potentially involves changes in the activation energy of the reaction as well as the thermodynamic properties of enzymes, *E*_Δ*H*_, *E*_Δ*Cp*_, and *T*_*m*_.

**Figure 2:**
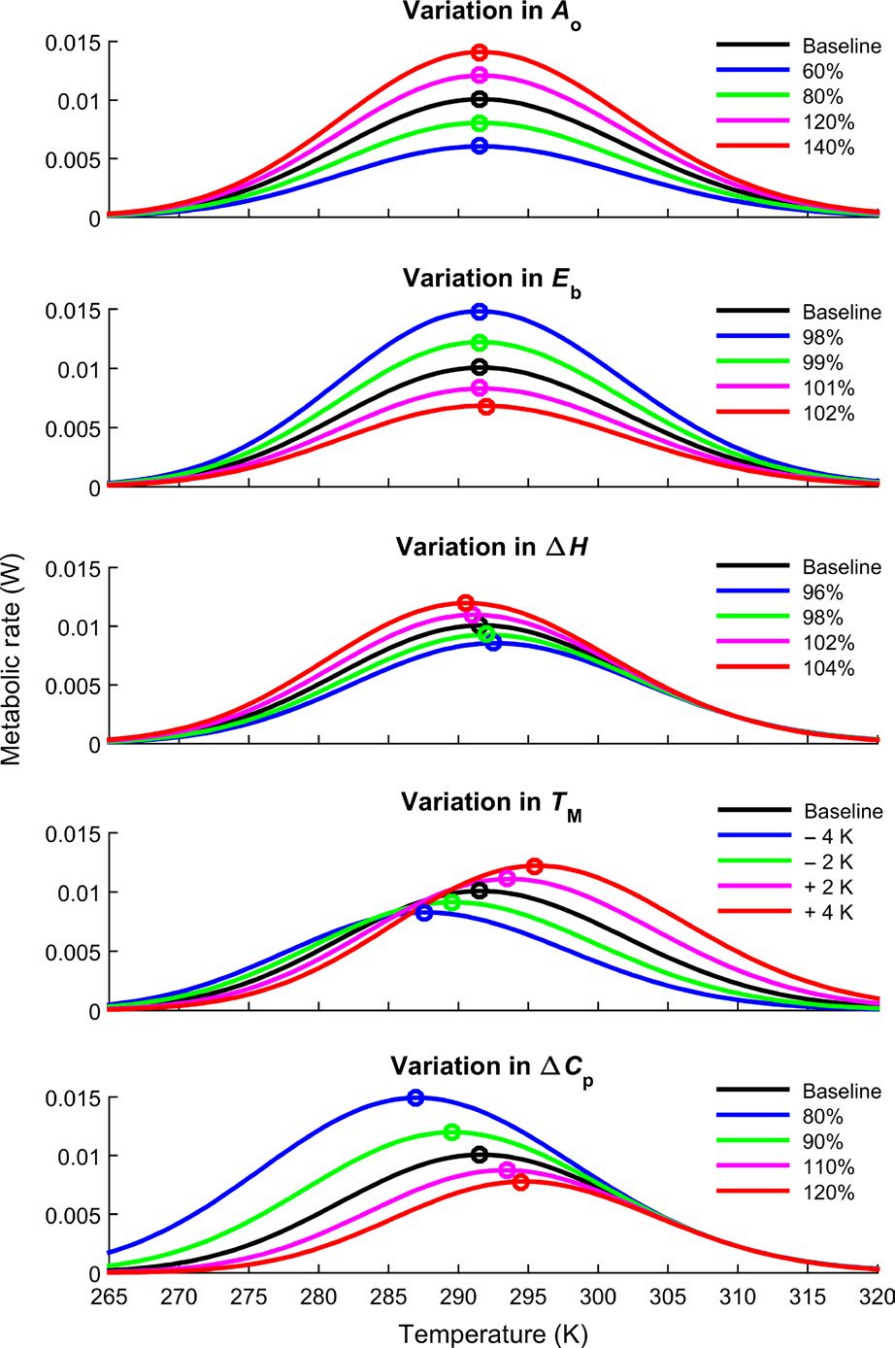
Effect of parameter variation on metabolic rate TPCs. Black is a representative TPC with parameter values similar to those derived from data on the amphipod *Niphargus verei* (Issartel et al., 2005): (Table S1). Other lines represent decreases (green and blue) or increases (magenta and red) in parameter values. With the exception of *A*_0,_ all parameters modify the *T*_opt_ (open circle) of metabolic rate, although the effect of *E*_*b*_ is very small

The EAAR model generates clear predictions about the acclimation and adaptation of metabolic rate TPCs to match environmental conditions (Figure 2). For example, Δ*C*_*p*_—the difference in heat capacity between the folded and unfolded state of enzymes—directly affects the spread or breadth of the TPC, so one clear route to becoming a thermal generalist is to lower enzymatic Δ*C*_*p*_. The model predicts that lowering Δ*C*_*p*_ not only broadens the curve, but also elevates the curve, suggesting that we might not predict a specialist—generalist trade‐off for all TPCs. Enzymes with higher Δ*H* are predicted to elevate the TPC due to increases in the enzyme contribution to *E*_*b*_ and to shift *T*_opt_ to lower temperatures, while increases in *T*_*m*_ will lower the curve and move the *T*_opt_ to the right. Any potential genetic correlations among parameters, however, could constrain the options for acclimation or evolution of the curves with changing thermal environments (Ratkowsky et al., 2005).

## 4 How well does the EAAR model do in describing real metabolic rate data?

As an initial assessment of the model's ability to describe real data, we fit the model to metabolic rate TPCs for three species of amphipod (Issartel, Hervant, Voituron, Renault, & Vernon, 2005), a stonefly (Heiman & Knight, 1975), and zebra mussels (*Dreissena polymorpha*) acclimated to two different temperatures (5 and 25°C; Alexander & McMahon, 2004). We emphasize that this is not a test of the model but an illustration of its utility for describing metabolic rate TPCs correctly and understanding how underlying mechanisms lead to changes in the shape of TPCs. The TPC data were plotted as means with standard errors in the original sources. We extracted the data from the figures, converted the units to whole‐organism metabolic rate in watts (W) from oxygen consumption, and modeled the full data set by randomly drawing data for each temperature given the reported sample size and the mean and standard deviation of metabolic rate for that temperature. We then log‐transformed the metabolic rate data and fit the log‐transformed EAAR model to each data set using nonlinear regression in MATLAB. We conducted the fitting in two steps. In step (1), we identified the melting temperature (*T*_*m*_) using a fit of a quadratic function to the right side of the data. This step is essential because the model is defined with respect to *T*_*m*_, and otherwise attempting to fit *T*_*m*_ in the overall fitting process can provide poor estimates of both *T*_*m*_ and the remaining parameters. In step (2), we fit the EAAR model to the rest of the data with the *T*_*m*_ set at the value identified in step (1). For each modeled data set, we also calculated the *T*_opt_ using Equation (6). We repeated this process 1,000 times per data set and used these distributions to identify mean and 95% confidence intervals for the model parameters, *T*_*m*_, and *T*_opt_.

**Figure 3:**
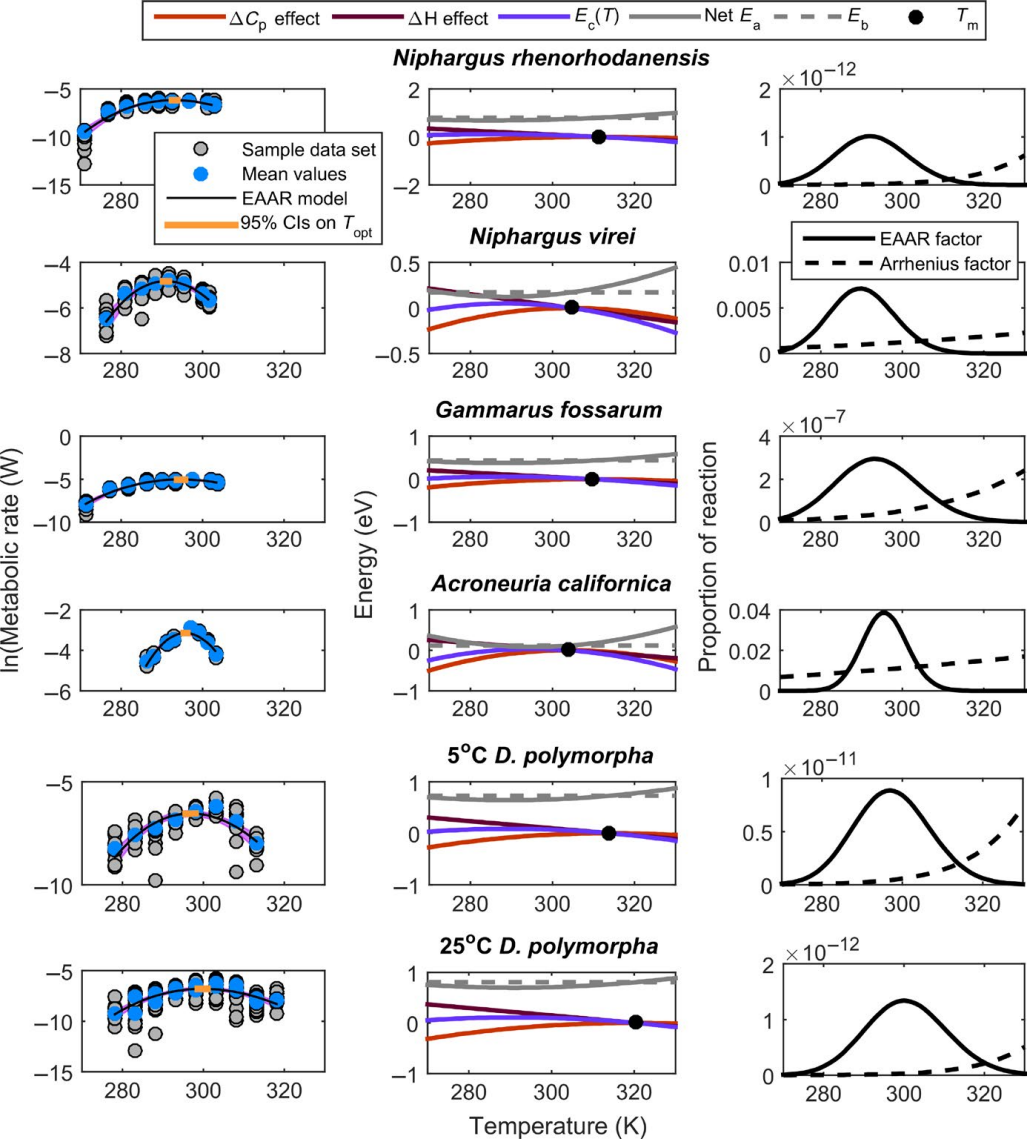
Left column. Thermal performance curves for metabolic rate with fits of the EAAR model for three amphipods: *Niphargus rhenorhodanensis, N. virei,* and *Gammarus fossarum* data from (Issartel et al., 2005), the stonefly *Acroneuria californica* (Heiman & Knight, 1975), and for zebra mussels (*Dreissena polymorpha*) acclimated to 5 and 25°C (Alexander & McMahon, 2004). Blue dots are means for each temperature as reported in the original source, and the gray dots are a sample of a simulated data set we used for fitting that has the same sample size as the original data set and is generated by randomly sampling from a normal distribution set by the reported mean and standard deviation. The orange bar is the 95% confidence intervals of *T*_opt_, calculated using equation (6), and the gray shaded region is the 95% confidence interval of the fit, from each of 1000 modeled data sets. Middle column. Model components and their effect on the kinetic hurdle of metabolic reactions (i.e., the *y*‐axis in Figure 1). The black dot is the melting temperature, *T*_*m*_. Right column. The proportion of the potential reaction that can occur given the temperature. The Arrhenius factor is the standard model, while the EAAR factor is the Arrhenius factor that considers the temperature dependence of enzyme stability. Fitted curves suggest that enzymatic properties are altered by acclimation in the zebra mussels so that both the melting (*T*_*m*_) and optimal temperatures (*T*_opt_) are higher when acclimated to higher temperatures

**Figure 4:**
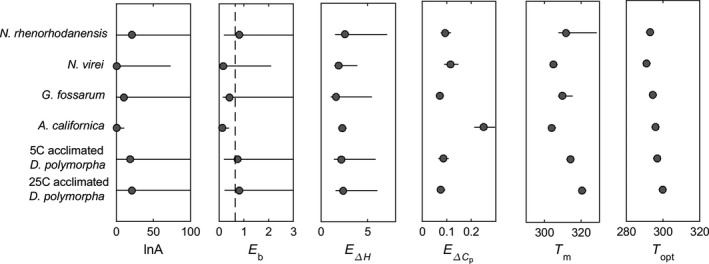
Parameter estimates and curve properties with 95% confidence intervals from fits of the EAAR model to TPC data from the three amphipods, the stonefly, and the zebra mussels shown in Figure 3. Dashed line shows 0.65 ev

The EAAR model captures the shape of the metabolic TPCs exceedingly well across all these organisms, including the rising and falling portions of the curves as well as the optimal temperature (Figure 3, first column, Table S1). Most of the parameters varied widely, but *E*_Δ*H*_ seemed somewhat conserved across TPCs (Figure 4, Table S1). The second column in Figure 3 shows how the components of the model interact to set the modified Arrhenius factor in the EAAR model (i.e., the EAAR factor). Heat capacity and enthalpy of formation interact to set the temperature‐dependent energetic contribution to the reaction, generating an upward opening *E*_*c*_. The model fits to the data also reveal that the baseline activation energy, *E*_*b*_, is in line with previous expectations of the observed activation energy, *E*_*a*_, of near .65 eV (confidence intervals overlap .65 in all but one case), although there is some variation among taxa (Table S1). The third column of Figure 3 shows the Arrhenius factor given only the reactant kinetic response. The enzymatic contributions are large compared to the kinetic contributions, driving the reaction up and over the thermal optima.

Because the EAAR model has a mechanistic derivation, parameter differences across data sets or conditions have biological meaning, although because these parameters are compound we should interpret them with some caution. Nonetheless, some parameter differences were evident among the data sets shown in Figure 3. For example, the amphipod *N. rhenorhodanensis* has a lower *E*_Δ*Cp*_ than the other amphipods, consistent with the higher thermal optimum and broader TPC for this species. In contrast, the stonefly has a much higher *E*_Δ*Cp*_, generating its narrow TPC. The zebra mussels acclimated to 5 and 25°C showed no shifts in the stability properties of the enzymes, but the *T*_*m*_ and *T*_opt_ were higher at the higher acclimation temperature (Figures 3; Table S1).

## 5 Discussion

There is a long history of work attempting to understand the temperature dependence of metabolic rate. The issues became somewhat controversial in the 2000s with the advent of MTE and opposing views (Allen & Gillooly, 2007; Brown et al., 2004; Clarke, 2004, 2006; Gillooly et al., 2006; Knies & Kingsolver, 2010; O'Connor et al., 2007). Important problems with the use of the Arrhenius equation included the model itself, because it neither shows a unimodal response to temperature nor allows for variation in activation energy across temperature. The Arrhenius equation also has been criticized as being not mechanistic, because it ascribes all of the temperature dependence of metabolism to kinetics and bypasses a wide range of physiological processes (Clarke, 2006). Despite the controversy, the Arrhenius equation has enabled considerable insights into thermal ecology, even as an understanding of the full response to temperature has remained unresolved.

Several attempts have been made to create models with a unimodal response of enzyme‐catalyzed reactions to temperature (Box 1). These models make some implausible assumptions, particularly that enzymes do not lower the reaction's activation energy (Assumption #12). Like the other models (Box 1), the EAAR model maintains the importance of reactant kinetics in driving metabolic rates, but it incorporates the more complex reality of how enzymes facilitate the reaction. Metabolism does not run by itself even in the biologically relevant temperature range—it requires enzymes to lower the kinetic hurdle. Enzymes modify the activation energy, which is why the observed activation energy should be understood as the kinetic hurdle that remains after enzymes have done their job (*E*_*b*_ − *E*_*c*_). The EAAR model uses the thermodynamics of protein stability to describe how enzymes increase and then decrease in effectively lowering the activation energy as temperatures rise, and the model describes well the dependence of metabolic rate on temperature for diverse organisms (Figures 3).

The EAAR model is built on a mechanistic derivation of protein stability in which all the parameters have thermodynamic meaning. However, we do not know whether this reflects the thermal dependence of a single key protein (e.g., a rate‐limiting step), the sharing of similar thermal dependencies of the many enzymes that underlie metabolic rate within an organism, or whether it is the average of many enzymes which share control of metabolism, each of which may differ somewhat in their thermal optima (Darveau, Suarez, Andrews, & Hochachka, 2002). The standard interpretation in MTE is that the observed activation energy (*E*_*a*_) for metabolic rates represents an average activation energy for the rate‐limiting enzyme‐catalyzed biochemical reactions that govern metabolism (Gillooly et al., 2001, 2006). We recognize that our model obscures some of the underlying physiological mechanisms driving metabolic rate by focusing on the net outcome of many individual reactions operating within a complex system of biochemical networks and structures. For example, while the downward slope of the TPC in multicellular organisms may be explained by the thermal dependence of enzyme Δ*G*, it may also be the result of failure at higher levels of biological organization (e.g., neural processes, membrane‐associated functions, mitochondrial failure or any number of processes that affect oxygen and energy supply and demand). Protein stability may be modified by extrinsic changes in pH, thermoprotectant osmolytes, protein concentration, and, in the case of membrane‐localized proteins, the membrane architecture. Additionally, a variety of stressors including temperatures near and above thermal optima activate the heat‐shock protein response (Verghese, Abrams, Wang, & Morano, 2012) or the signals that promote the production of heat‐shock proteins (Kaspari et al., 2016). Finally, although we did not observe this in the data sets compared here, regulation of substrate availability (*A*_0_) via regulation of flux in response to temperature may be a critical component of thermal responses of metabolic rate (Schulte, 2015; Suarez & Moyes, 2012). These mechanisms could modify the TPC beyond what could be expected from the EARR model (or any of the other models shown in Box 1). Thus, we do not argue that the EAAR model is a complete depiction of the processes that drive metabolic rate but a useful tool for understanding thermal ecology and predicting the consequences of changes in temperature on organism performance.

In addition, the model makes useful connections between whole‐organism rates and underlying mechanisms by building on fundamental, thermodynamic aspects of all protein stability curves (Feller, 2010). Thus, our model reveals potential mechanistic links between individual reactions and whole‐organism rates which can serve as hypotheses about climate adaptation and point toward additional research. In the case of the zebra mussels (Figures 3, 4), acclimation to warmer temperatures involved a change in metabolic processes to be more stable at high temperatures (increased *T*_*m*_ and *T*_opt_), but other parameters, such as substrate levels (*A*_0_), *E*_Δ*H*_, and *E*_Δ*Cp*_ were unchanged. One possibility for such changes is that acclimation of mitochondrial membranes to temperature may be a factor determining the thermal stability of the membrane‐embedded protein oxidative phosphorylation complexes, which may play a critical role in how thermal acclimation shapes TPCs for aerobic metabolic rate (Dahlhoff & Somero, 1993; Gibbs & Somero, 1990; Weinstein & Somero, 1998).

The molecular evolution underlying divergence in intrinsic protein stability across the range of temperatures inhabited by life appears to be shaped by a common set of thermodynamic rules that govern protein folding (Feller, 2010; Hochachka & Somero, 2002). Nonetheless, adaptive molecular changes in protein conformational thermostability involve diverse amino acid substitutions that can affect the strength of noncovalent interactions, the binding of stabilizing ions, the surface charges of the molecule, or modify conformational entropy (Feller, 2010; Hochachka & Somero, 2002). The EAAR model can therefore help link specific pathways of molecular evolution to whole‐organism function via the parameters that reflect thermostability, and as such generates new opportunity to provide insight into adaptation to different thermal environments.

The right column of Figure 3 suggests a surprising response of metabolic rate to temperature. Our data and model suggest that the rise and fall of the EAAR factor comes mostly from an increase and decrease in the enzymatic contributions to the reaction, as the activation hurdle drops and then rises again. The right side of this curve is anchored at the melting temperature, *T*_*m*_, where by definition the enzymes are no longer contributing to the reaction and the organism is near death. Above this point, however, the reactant kinetic effect of temperature is still increasing, which suggests that the remaining reactants present in an organism that is pushed above this temperature should react at a faster rate, even if the organism has died, at least until the reactants decline in availability. Surprisingly, this outcome has been observed in what is known as thermolimit respirometry, where the metabolic rate of an organism is measured as the temperature is ramped up, and an increase in the rate of metabolism is observed after the organism dies (Lighton, 2007). Although we do not claim that the good fits to data we obtained above should be treated as tests of the EAAR model, the model's novel prediction of an uptake in metabolic rate above the melting temperature was made independent of data, providing an unintended qualitative test. No other metabolic rate TPC model that we know of predicts this aspect of thermolimit respirometry (Box 1).

In summary, the EAAR model captures the empirical patterns and incorporates the minimum necessary processes shaping the temperature dependence of metabolic rate in a relatively simple and useful manner. It resolves long‐term problems with the Arrhenius equation and other reaction models and provides a way to move forward with temperature in metabolic ecology. In particular, the EAAR model will facilitate more comparative analyses to elucidate mechanisms involved in acclimation and adaptation to different thermal environments. Furthermore, the many biological processes that depend on metabolic rate can be understood as extensions of the EAAR process. For example, the EAAR model should apply equally well to photosynthesis, which also shows a unimodal response to temperature (Padfield, Yvon‐Durocher, Buckling, Jennings, & Yvon‐Durocher, 2015). In addition, the role of metabolic rate in driving other processes such as locomotion can be captured by incorporating the EAAR model into biomechanical models of movement to understand the unimodal temperature dependence of animal movement and the consequences of warming on these processes (Gibert et al., 2016). Similarly, the role of metabolism in driving production and population growth can incorporate the EAAR model to capture and understand the responses of population processes to a wider range of temperatures. This will be increasingly important, as climate variation across unimodal responses to temperature are critical to predicting future organism performance (Deutsch et al., 2008; Vasseur et al., 2014), and TPCs can change quickly as ecological conditions change (Kingsolver, Massie, Ragland, & Smith, 2007; Luhring & DeLong, 2016), indicating an immediate need for understanding and predicting the shape of TPCs.

## Conflict of Interest

The authors declare no conflict of interest.

## Data Accessibility

The data used in this article are previously published and available in the original sources. Our digitization of the data will be made available on Data Dryad upon acceptance of the manuscript.

## Author Contributions

All authors were involved in the conception of the paper and contributed to the collection of data. JPD and JPG developed the model. JPD and TML analyzed the data. All authors contributed critically to the drafts and figures and gave final approval for publication.
