# Supplementary material for: The combined effects of reactant kinetics and enzyme stability explain the temperature dependence of metabolic rates
Source: Ecol Evol. 2017 Apr 23;7(11):3940–50. doi: 10.1002/ece3.2955 (PMC5468145; doi:10.1002/ece3.2955)
Supplement: Supplementary file 1 [file ECE3-7-3940-s001.docx]

**The combined effects of reactant kinetics and enzyme stability explain the temperature dependence of metabolic rates**

J.P. DeLong, J.P. Gibert, T.M. Luhring, G. Bachman, B. Reed, A. Neyer, K.L. Montooth

Figure S1. **A**. Plot of the Arrhenius factor ($e^{\frac{-E_{a}}{kT}}$) – the proportion of the potential reaction rate *A*_0_ that can occur given the kinetic state of the reactants – over a wide temperature range, showing the rapid rise at lower temperatures and a tapering off at very high temperatures. **B**. The Arrhenius factor over a narrower range, showing that the factor is extremely small over the biologically relevant temperature range. At 37°C (i.e., normal human body temperature) the Arrhenius equation predicts that only about 10^-9^% of the potential reaction rate given by *A*_0_ can occur. This suggests that metabolic rates should be vanishingly low in the biologically relevant temperature range, and only with a massive supply of substrates can the reaction proceed.


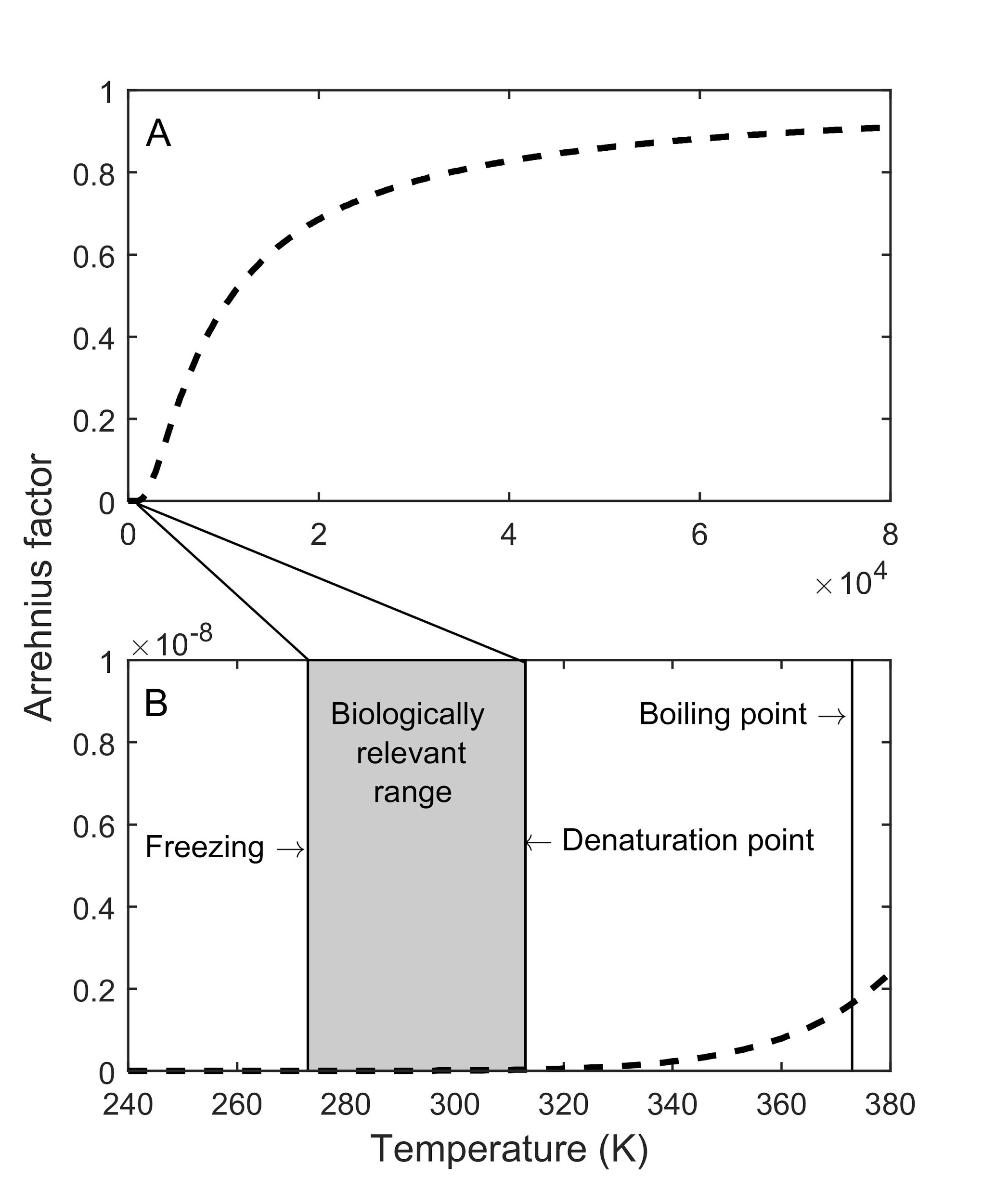


Table S1. Parameters and CIs for fits to data sets.

| **Source** | **Taxon** | **Scientific name** | **Median *R*^2^** | **ln*A*** | **95% down** | **95% up** | ***E*_b_** | **95% down** | **95% up** | ***E*_Δ_*_H_*** | **95% down** | **95% up** |
| --- | --- | --- | --- | --- | --- | --- | --- | --- | --- | --- | --- | --- |
| Issartel, et al, 2005 | Amphipod | *Nephargus verei* | 0.85 | 20.85 | 0.004 | 157.3 | 0.80 | 0.20 | 4.54 | 2.59 | 1.53 | 7.07 |
| Issartel, et al, 2005 | Amphipod | *Gammarus fossarum* | 0.66 | 0.26 | 0.019 | 72.7 | 0.17 | 0.16 | 2.08 | 1.88 | 1.44 | 3.85 |
| Issartel, et al, 2005 | Amphipod | *Nephargus rhenorhodanensis* | 0.89 | 10.10 | 0.002 | 152.4 | 0.43 | 0.16 | 4.24 | 1.60 | 1.06 | 5.42 |
| Heiman and Knight 1975 | Stonefly | *Acroneuria californica* | 0.82 | 0.17 | 0.015 | 10.2 | 0.12 | 0.11 | 0.38 | 2.26 | 1.90 | 2.69 |
| Alexander Jr. and McMahon, 2004 | Zebra mussel | *Dreissena polymorpha*, 5°C | 0.54 | 18.79 | 0.008 | 150.3 | 0.73 | 0.21 | 4.29 | 2.17 | 1.42 | 5.81 |
| Alexander Jr. and McMahon, 2021 | Zebra mussel | *Dreissena polymorpha*, 25°C | 0.39 | 20.62 | 0.002 | 150.8 | 0.81 | 0.24 | 4.40 | 2.33 | 1.60 | 5.99 |

| **Source** | **Scientific name** | ***E*_Δ_*_C_*_p_** | **95% down** | **95% up** | ***T*_m_** | **95% down** | **95% up** | ***T*_opt_** | **95% down** | **95% up** |
| --- | --- | --- | --- | --- | --- | --- | --- | --- | --- | --- |
| Issartel, et al, 2005 | *Nephargus verei* | 0.09 | 0.08 | 0.12 | 311.5 | 307.8 | 328.3 | 293.0 | 292.4 | 293.8 |
| Issartel, et al, 2005 | *Gammarus fossarum* | 0.11 | 0.09 | 0.15 | 304.7 | 303.7 | 306.6 | 291.0 | 290.3 | 291.7 |
| Issartel, et al, 2005 | *Nephargus rhenorhodanensis* | 0.07 | 0.06 | 0.08 | 309.8 | 307.7 | 315.1 | 294.6 | 293.8 | 295.7 |
| Heiman and Knight 1975 | *Acroneuria californica* | 0.25 | 0.21 | 0.30 | 304.0 | 303.7 | 304.5 | 295.8 | 295.4 | 296.2 |
| Alexander Jr. and McMahon, 2004 | *Dreissena polymorpha*, 5°C | 0.08 | 0.07 | 0.11 | 314.0 | 313.5 | 314.7 | 296.9 | 295.6 | 298.1 |
| Alexander Jr. and McMahon, 2021 | *Dreissena polymorpha*, 25°C | 0.08 | 0.06 | 0.09 | 320.3 | 319.3 | 321.5 | 299.8 | 298.7 | 301.0 |

Matlab code for data simulation and analysis:

clear; clc; clf;

[num text] = xlsread('TPC_data_set_theory_paper','Metabolic data');

% read in columns

temp_all = num(:,8)+273.15;

MR_all = num(:,17);

MR_SE_all = num(:,18);

for z = 1:6 % run for 6 data sets

if z == 1 % Niphargus rhenorhodanensis

species = '\itNiphargus rhenorhodanensis';

temp = temp_all(1:9);

mr = MR_all(1:9);

mr_SE = MR_SE_all(1:9);

sample_size = 10;

elseif z == 2 % Niphargus virei

species = '\itNiphargus virei';

temp = temp_all(10:17);

mr = MR_all(10:17);

mr_SE = MR_SE_all(10:17);

sample_size = 10;

elseif z == 3 % Gammarus fossarum

species = '\itGammarus fossarum';

temp = temp_all(18:26);

mr = MR_all(18:26);

mr_SE = MR_SE_all(18:26);

sample_size = 10;

elseif z == 4 % Acroneuria californica

species = '\itAcroneuria californica';

temp = temp_all(44:51);

mr = MR_all(44:51);

mr_SE = MR_SE_all(44:51);

sample_size = 3;

elseif z == 5 % 5C acclimated Dreissena polymorpha

species = '5C acclimated \itDreissena polymorpha';

temp = temp_all(27:34);

mr = MR_all(27:34);

mr_SE = MR_SE_all(27:34);

sample_size = 12;

elseif z == 6 % 25C acclimated Dreissena polymorpha

species = '25C acclimated \itDreissena polymorpha';

temp = temp_all(28:43);

mr = MR_all(28:43);

mr_SE = MR_SE_all(28:43);

sample_size = 12;

end

% data transformations

lnmr = log(mr);

mr_SD = mr_SE.*sqrt(sample_size); % calculate SE from SD

for i = 1:length(mr) % create modeled data temp vector

temp_matrix(1:sample_size,i) = temp(i);

end

temp_mod = reshape(temp_matrix,[sample_size*length(temp),1]); % turn matrix into column vector

num_bootstraps = 1000; % how many bootstrapped data sets to use

rsquares = NaN(num_bootstraps,1); % open an empty vector to put in rsquareds

parameters = NaN(num_bootstraps,4); % open an empty vector to put in parameters

topt = NaN(num_bootstraps,1); % open an empty vector for topt

tref = NaN(num_bootstraps,1); % open an empty vector for tref

num_fits = 0; % start fitting index at 0

for j = 1:1000 % start bootstrap

if num_fits < num_bootstraps+1 % keep fitting if you haven't done them all

% create modeled data set

for i = 1:length(mr)

mr_matrix(:,i) = mr(i) + mr_SD(i).*randn(sample_size,1); % generate data for each temperature

end

mr_mod = reshape(mr_matrix,[sample_size*length(temp),1]); % reshape data set for fitting

lnmr_mod = log(mr_mod); % lake log

% find tref

index = (find(mr == max(mr))-1):length(mr); % find right side of TPC

temp_tref = temp_matrix(:,index); % temp for right side based on above index

temp_tref_X = reshape(temp_tref,[sample_size*length(index),1]);

mr_tref = mr_matrix(:,index); % mr for right side based on above index

mr_tref_X = reshape(mr_tref,[sample_size*length(index),1]);

ft_ref = fittype( 'poly2' ); % set up fit as second order polynomial

opts = fitoptions( 'Method', 'LinearLeastSquares' ); % choose method

[fitresult1, gof1] = fit( temp_tref_X, mr_tref_X, ft_ref, opts ); % Fit model to data

% figure( 'Name', 'untitled fit' ); % plot fit with data (best to leave this commented unless exploring)

% h = plot( fitresult1, temp_tref, mr_tref );

% xlabel tempk

% ylabel mr

fit_roots = roots(coeffvalues(fitresult1)); % find roots of fitted polynomial

tref(j) = max(real(fit_roots)); % take real parts where necessary

trefs = num2str(tref(j)); % switch to string to include below

% fit real TPC curve

ft = fittype(['lnA - (Ea - deltaH*(1 - x/',trefs,') - deltaCp*(x - ',trefs,' - x*log(x/',trefs,')))/(x*8.617e-5)'], 'independent', 'x', 'dependent', 'y' );

opts = fitoptions( ft ); % Set up options

opts.Display = 'Off';

opts.Lower = [-Inf 0 -Inf 0];

opts.MaxFunEvals = 6000000;

opts.MaxIter = 4000000;

opts.StartPoint = [0.1 1 0.001 0.01];

opts.Upper = [Inf Inf Inf Inf];

try

[fitresult, gof] = fit( temp_mod, lnmr_mod, ft, opts );

catch ME

end

rsquares(j) = gof.rsquare; % log the rsquare value

num_fits = num_fits + 1; % index the fitting

coeffs = coeffvalues(fitresult); % pull out the fitted parameters

parameters(j,:) = coeffs; % log them

overall_curve = feval(fitresult,temp_mod); % calculate the fitted curve for this bootstrap

temps_for_fit = min(temp_mod):1:max(temp_mod); % set temperatures

curve2(j,:) = feval(fitresult,temps_for_fit)'; % calculate the fitted curve for the set temps

topt(j) = (coeffs(1) - coeffs(3) + coeffs(2)*tref(j))/coeffs(2); % calculate topt

curve2(curve2(:,1)==0,:) = [];

% figure(2);clf(2);

% box on; hold on;

% plot(temp_mod,lnmr_mod,'ok','MarkerFaceColor',[0.5 0.5 0.5]);

% plot(temp_mod,overall_curve,'-k','LineWidth',2);

end

end

topt(topt(:,:)==0) = []; % clear out bad fits with zeros

curve_up = prctile(curve2(:,:),97.5); % pull out curves from bootstrapped fits

curve_down = prctile(curve2(:,:),2.5);

curve_med = prctile(curve2(:,:),50);

% compile parameters with confidence intervals and print to excel sheet

lnA = prctile(parameters(:,4),50);

lnA_97_5 = prctile(parameters(:,4),97.5);

lnA_2_5 = prctile(parameters(:,4),2.5);

Ea = prctile(parameters(:,1),50);

Ea_97_5 = prctile(parameters(:,1),97.5);

Ea_2_5 = prctile(parameters(:,1),2.5);

deltaH = prctile(parameters(:,3),50);

deltaH_97_5 = prctile(parameters(:,3),97.5);

deltaH_2_5 = prctile(parameters(:,3),2.5);

deltaCp = prctile(parameters(:,2),50);

deltaCp_97_5 = prctile(parameters(:,2),97.5);

deltaCp_2_5 = prctile(parameters(:,2),2.5);

tref_med = prctile(tref,50);

tref_97_5 = prctile(tref,97.5);

tref_2_5 = prctile(tref,2.5);

topt_med = prctile(topt,50);

topt_97_5 = prctile(topt,97.5);

topt_2_5 = prctile(topt,2.5);

% find closest part of median curve to plot ci's on topt

length(temps_for_fit)

for q = 1:length(temps_for_fit)

val = temps_for_fit(q) %value to find

tmp_low(q) = abs(topt_2_5-val)

tmp_hi(q) = abs(topt_97_5-val)

end

[idx idx] = min(tmp_low); %index of closest value

lnmr_med_low = curve_med(idx); %closest value

[idx idx] = min(tmp_hi); %index of closest value

lnmr_med_hi = curve_med(idx); %closest value

parameters_out(z,:) = [median(rsquares) lnA lnA_2_5 lnA_97_5 Ea Ea_2_5 Ea_97_5 deltaH deltaH_2_5 deltaH_97_5 deltaCp deltaCp_2_5 deltaCp_97_5,...

tref_med tref_2_5 tref_97_5 topt_med topt_2_5 topt_97_5];

figure(2);

subplot(6,3,z*3-2);

box on;

jbfill(min(temp_mod):1:max(temp_mod),curve_up,curve_down,[0.69 0 1],[0.69 0 1],0.5,0.5); hold on;

l1 = plot(temp_mod,lnmr_mod,'ok','MarkerFaceColor',[0.7 0.7 0.7]);

l2 = plot(temp,lnmr,'o','MarkerEdgeColor',[0 0.53 1],'MarkerFaceColor',[0 0.53 1]);

l3 = plot(temps_for_fit,curve_med,'-k','LineWidth',1);

l4 = plot([topt_2_5 topt_97_5],[lnmr_med_low lnmr_med_hi],'-','Color',[1 0.6 0.2],'LineWidth',3);

xlim([270 330]);

if z == 1

legend([l1 l2 l3 l4],'Sample data set','Mean values','EAAR model','95% CIs on {\itT}_{opt}',...

'Location','SouthEast');

elseif z == 4

ylabel( 'ln(Metabolic rate (W))','FontSize',12);

end

temps_for_effects = 270:330;

deltaH_effect = deltaH.*(1 - temps_for_effects./tref_med);

deltaCp_effect = deltaCp.*(temps_for_effects - tref_med - temps_for_effects.*log(temps_for_effects./tref_med));

Ec_of_T = deltaH_effect + deltaCp_effect;

net_Ea = Ea - (Ec_of_T);

eaar_factor = exp(-(net_Ea)./(temps_for_effects.*8.617e-5));

arrhenius_factor = exp(-(Ea)./(temps_for_effects.*8.617e-5));

subplot(6,3,z*3-1);

box on; hold on;

h1 = plot(temps_for_effects,deltaCp_effect,'-','Color',[0.8 0.2 0],'LineWidth',2);

h2 = plot(temps_for_effects,deltaH_effect,'-','Color',[0.4 0 0.2],'LineWidth',2);

h3 = plot(temps_for_effects,Ec_of_T,'-','Color',[0.4 0.2 1],'LineWidth',2);

h4 = plot(temps_for_effects,net_Ea,'-','LineWidth',2,'Color',[0.5 0.5 0.5]);

h5 = plot([min(temps_for_effects) max(temps_for_effects)],[Ea Ea],'--','LineWidth',2,'Color',[0.5 0.5 0.5]);

h6 = plot(tref_med,0,'ok','MarkerFaceColor','k');

xlim([270 330]);

title(species);

if z == 1

legend([h1 h2 h3 h4 h5 h6],'\DeltaCp effect','\DeltaH effect','{\itE}{_c}({\itT})','Net {\itE}{_a}',...

'{\itE}{_b}','{\itT}_{m}','Location','SouthOutside');

elseif z == 4

ylabel( 'Energy (ev)','FontSize',12);

elseif z == 7

xlabel( 'Temperature (K)','FontSize',12);

end

subplot(6,3,z*3);

box on; hold on;

g1 = plot(temps_for_effects,eaar_factor,'-k','LineWidth',2);

g2 = plot(temps_for_effects,arrhenius_factor,'--k','LineWidth',2);

xlim([270 330]);

if z == 2

legend([g1 g2],'EAAR factor','Arrhenius factor','Location','NorthEast');

elseif z == 4

ylabel( 'Proportion of reaction','FontSize',12);

end

% clear these variables as they need to be reused and set at new sizes

% for each data set

clear('temp_matrix')

clear('mr_matrix')

clear('temp_mod')

clear('temps_for_fit')

clear('curve2')

clear('tmp_hi')

clear('tmp_low')

figure(3); % figure to plot parameter estimates and CIs

plot_index = -1*z+7;

subplot(1,6,1);

hold on; box on;

plot([lnA_2_5 lnA_97_5],[plot_index plot_index],'-k');

plot(lnA,plot_index,'ok','MarkerFaceColor',[0.5 0.5 0.5]);

xlabel('lnA');

xlim([0 100]);

ylim([0 7]);

set(gca,'YTick',[]);

subplot(1,6,2);

hold on; box on;

plot([Ea_2_5 Ea_97_5],[plot_index plot_index],'-k');

plot(Ea,plot_index,'ok','MarkerFaceColor',[0.5 0.5 0.5]);

xlabel('{\itE}{_b}');

xlim([0 3]);

ylim([0 7]);

set(gca,'Ytick',[]);

subplot(1,6,3);

hold on; box on;

plot([deltaH_2_5 deltaH_97_5],[plot_index plot_index],'-k');

plot(deltaH,plot_index,'ok','MarkerFaceColor',[0.5 0.5 0.5]);

xlabel('\DeltaH');

xlim([0 8]);

ylim([0 7]);

set(gca,'Ytick',[]);

subplot(1,6,4);

hold on; box on;

plot([deltaCp_2_5 deltaCp_97_5],[plot_index plot_index],'-k');

plot(deltaCp,plot_index,'ok','MarkerFaceColor',[0.5 0.5 0.5]);

xlabel('\DeltaCp');

xlim([0 0.3]);

ylim([0 7]);

set(gca,'Ytick',[]);

subplot(1,6,5);

hold on; box on;

plot([tref_2_5 tref_97_5],[plot_index plot_index],'-k');

plot(tref_med,plot_index,'ok','MarkerFaceColor',[0.5 0.5 0.5]);

xlabel('{\itT}_m');

xlim([290 330]);

ylim([0 7]);

set(gca,'Ytick',[]);

subplot(1,6,6);

hold on; box on;

plot([topt_2_5 topt_97_5],[plot_index plot_index],'-k');

plot(topt_med,plot_index,'ok','MarkerFaceColor',[0.5 0.5 0.5]);

xlabel('{\itT}_{opt}');

xlim([280 320]);

ylim([0 7]);

set(gca,'Ytick',[]);

end

figure(3);subplot(1,6,2); plot([0.65 0.65],[0 7],'--k');

gtext('\itN. rhenorhodanensis')

gtext('\itN. virei')

gtext('\itG. fossarum')

gtext('\itA. californica')

gtext({['5C acclimated'],['\itD. polymorpha']})

gtext({['25C acclimated'],['\itD. polymorpha']})
